# Zhilong Huoxue Tongyu Capsule Alleviated the Pyroptosis of Vascular Endothelial Cells Induced by ox-LDL through miR-30b-5p/NLRP3

**DOI:** 10.1155/2022/3981350

**Published:** 2022-01-27

**Authors:** Mengnan Liu, Gang Luo, Tianzhu Liu, Tingfu Yang, Raoqiong Wang, Wei Ren, Ping Liu, Xiaoling Lai, Hua Zhou, Sijin Yang

**Affiliations:** ^1^National Traditional Chinese Medicine Clinical Research Base and Department of Cardiovascular, Hospital (T.C.M) Affiliated to Southwest Medical University, Luzhou, Sichuan, China; ^2^Faculty of Chinese Medicine and State Key Laboratory of Quality Research in Chinese Medicine, Macau University of Science and Technology, Taipa, Macao, China; ^3^Department of Neurology, Hospital (T.C.M) Affiliated to Southwest Medical University, Luzhou, Sichuan, China; ^4^Department of Scientific Research, Hospital (T.C.M) Affiliated to Southwest Medical University, Luzhou, Sichuan, China; ^5^Drug Research Center of Integrated Traditional Chinese and Western Medicine, Hospital (T.C.M) Affiliated to Southwest Medical University, Luzhou, Sichuan, China; ^6^Zhuhai Hospital of Integrated Traditional Chinese and Western Medicine, Zhuhai, Guangdong, China; ^7^Joint Laboratory for Translational Cancer Research of Chinese Medicine of the Ministry of Education of the People's Republic of China, Guangzhou University of Chinese Medicine, Guangzhou, Guangdong, China

## Abstract

**Background:**

Our previous studies have demonstrated a protective role of Zhilong Huoxue Tongyu capsule in atherosclerosis (AS); however, the molecular mechanisms are unclear.

**Methods:**

Human coronary artery endothelial cells (HCAECs) were induced with oxidized low-density lipoprotein (ox-LDL) to obtain cellular AS models. Then, the medicated serum of Zhilong Huoxue Tongyu capsule was obtained and used for treatment with ox-LDL-induced HCAECs. The cell viability was detected by CCK-8 assay. Besides, the binding between miR-30b-5p and NLRP3 was determined by the dual-luciferase reporter gene system assay. Furthermore, ox-LDL-induced HCAECs were transfected with miR-30b-5p mimic or miR-30b-5p inhibitor. The pyroptosis of HCAECs was assessed by flow cytometry, LDH content detection, and qRT-PCR assays.

**Results:**

10% medicated serum of Zhilong Huoxue Tongyu capsule was the maximum nontoxic concentration and it was used in subsequent assays. The rate of pyroptosis, LDH content, and the mRNA expression level of pyroptosis-related genes including NLRP3, ASC, Caspase 1, IL-1*β*, and IL-18 were prominently enhanced after HCAECs were induced by ox-LDL, which were markedly rescued with medicated serum of Zhilong Huoxue Tongyu capsule. In addition, the medicated serum of Zhilong Huoxue Tongyu capsule significantly enhanced the ox-LDL-induced reduction of miR-30b-5p level. NLRP3 could bind to miR-30b-5p and was negatively corrected with miR-30b-5p. Moreover, all the rates of pyroptosis, LDH content, and the mRNA expression levels of pyroptosis-related genes including NLRP3, ASC, Caspase 1, IL-1*β*, and IL-18 were further observably decreased after ox-LDL-induced HCAECs treated with medicated serum were transfected with miR-30b-5p mimic, while these were significantly rescued with transfection of miR-30b-5p inhibitor.

**Conclusion:**

Zhilong Huoxue Tongyu capsule alleviated the pyroptosis of vascular endothelial cells induced by ox-LDL through miR-30b-5p/NLRP3.

## 1. Introduction 

Atherosclerosis (AS) is the main cause of a variety of cardiovascular and cerebrovascular diseases, including coronary heart disease and stroke, which has brought about huge medical and economic burdens to society and individuals [1]. The occurrence and development of AS involve multiple factors. Among them, pathological factors such as smoking, hypertension, hyperlipidemia, hyperuric acid, and hyperglycemia causing vascular endothelial damage through inflammatory and oxidative stress responses are the initiating link of AS [2–4]. Thus, AS is a chronic inflammatory progress that is characterized by atherosclerotic plaques, vascular stenosis, inflammation, and lipid metabolism [5, 6]. Oxidative low-density lipoprotein (ox-LDL) plays a key role in atherosclerotic plaque formation and endothelial cell damage. After endothelial damage caused by ox-LDL, it can promote a variety of inflammatory cells to infiltrate the damaged vascular endothelium. Also, foam cells are formed and gradually accumulate to form atherosclerotic plaques after inflammatory cells engulf ox-LDL [7, 8]. Cell death caused by inflammation is closely related to cell pyroptosis [9]. Therefore, protecting endothelial cell pyrolysis caused by ox-LDL is considered to be a key target for treating AS and reducing the incidence and mortality of cardiovascular and cerebrovascular diseases.

Pyroptosis is a highly proinflammatory programmed cell death. Activated caspases cleave the pore-forming protein Gasdermin D (GSDMD) to rupture the plasma membrane, leading to the release of proinflammatory factors interleukin-1*β* (IL-1*β*) and IL-18 [10]. The assembly of the inflammasome is the first step in the beginning of cell pyrolysis, of which the inflammasome, such as NLRP3 inflammasome, binds to pattern recognition receptors to complete assembly [11]. Moreover, NLRP3 inflammasomes have been demonstrated to play an important role in the occurrence and development of AS. Duewell et al. reported that cholesterol crystals activate NLRP3 inflammasomes to promote pyroptosis of vascular endothelial cells, which leads to endothelial dysfunction to induce the occurrence of AS [12]. Furthermore, microRNAs (miRNAs), a category of endogenous, evolutionarily conserved, and small noncoding RNA sequences [13, 14], have been found to regulate the progress of pyroptosis via targeting miRNAs [15, 16]. Thus, the pyroptosis of vascular endothelial cells may be modulated via the miRNAs/NLRP3 signaling axis, which exerts a decisive and important role in the occurrence and development of AS.

Zhilong Huoxue Tongyu capsule is a pure Chinese medicine in-hospital preparation developed by Professor Yang Sijin of our hospital which is mainly developed from traditional Chinese medicines including *Astragalus*, *Pheretima*, Caulis Sargentodoxae, Cassia Twig, and *Hirudo*. Our group has demonstrated the outstanding effectiveness of Zhilong Huoxue Tongyu capsule on the ischemic stroke and related diseases, and its mechanism of action may be related to lowering blood lipids and inhibiting inflammation [17–19]. In addition, we also report that Zhilong Huoxue Tongyu capsule can improve the metabolism of serum lipids and reduce the formation of carotid atherosclerotic plaques in hyperlipidemia and carotid atherosclerosis rabbits, which may be associated with the inhibition of NLRP3 inflammasome activation and downstream inflammatory factors release by suppressing the phosphorylation of NF-*κ*B. Thus, we concluded that Zhilong Huoxue Tongyu capsule exhibited the antiatherosclerosis effect through NLRP3 inflammasome signaling pathway [20]. More importantly, recent study has shown that miR-30b-5p is negatively correlated with NLRP3 in mouse and human liver, and treatment of miR-30b-5p agomir can target NLRP3 and relieve liver inflammation in the injured liver [21]. Taken together, we speculated that the medicated serum of Zhilong Huoxue Tongyu capsule alleviated the pyroptosis of vascular endothelial cells induced by ox-LDL through miR-30b-5p/NLRP3.

The present study reports observable effectiveness of Zhilong Huoxue Tongyu capsule on the pyroptosis of vascular endothelial cells induced by ox-LDL in vitro. Mechanistically, Zhilong Huoxue Tongyu capsule alleviated the pyroptosis of vascular endothelial cells induced by ox-LDL through miR-30b-5p/NLRP3. The results of this study will provide new insights and methods for the therapy of AS and even other inflammation-related cardiovascular and cerebrovascular diseases.

## 2. Materials and Methods

### 2.1. Animal

Adult Sprague Dawley rats (age: 7-8 weeks, weight: 200–220 g) were purchased and acclimated to standard laboratory conditions for 7 days before experiments. Rats were provided with a 12 hour/12 h light-dark cycle and fed with standard diet and water ad libitum at (25 ± 2)°C and 40%–60% the relative humidity. All the procedures were carried out strictly based on the National Institute of Health Guide for the Care and Use of Laboratory Animals. Also, the study was ratified by the Board and Ethics Committee of Hospital (T.C.M) Affiliated to Southwest Medical University.

### 2.2. Cell Culture

Human coronary artery endothelial cells (HCAECs) were purchased from Procell (CP-H087, Wuhan, China) and cultured in complete medium for human umbilical vein endothelial cells (CM-H087, Wuhan, China) at 37°C with 5% carbon dioxide (CO_2_).

### 2.3. Cell Transfection

miR-30b-5p mimics, miR-30b-5p inhibitor, and the corresponding negative controls (NC) were designed and synthesized by Ribobio (Guangzhou, China). It was performed with RiboFect™ CP Transfection Kit (C10511-05, Ribobio) based on the experimental procedures for the following assays.

### 2.4. Preparation of Medicated Serum

For in vitro experiments, medicated serum was first prepared with the following description. 20 rats were randomly divided into two groups (*n* = 5): control and Zhilong Huoxue Tongyu capsule group. Rats in Zhilong Huoxue Tongyu capsule group were intragastrically administered with 2.52 g/(kg.d) Zhilong Huoxue Tongyu capsule, while rats in control group were intragastrically administered with 1 ml/100 g saline a day for 5 consecutive days. Then, blood was taken from the abdominal aorta after the rats were intraperitoneally anesthetized with sodium pentobarbital (40 mg/kg). Serum was isolated and the complement was inactivated for in vitro experiments.

### 2.5. Experimental Groups and Drug Administration

HCAECs were inoculated into six-well plates and divided into six groups: control, ox-LDL, ox-LDL + blank serum, ox-LDL + medicated serum (MS), ox-LDL + MS + miR-30b-5p-mimic, and ox-LDL + MS + miR-30b-5p-inhibitor. HCAECs in control group were cultured in complete medium for human umbilical vein endothelial cells, while cells in the other five groups were cultured in complete medium for human umbilical vein endothelial cells including 50 nM ox-LDL for 24 h [22]. Subsequently, cells in ox-LDL + blank serum group were cultured in complete medium for human umbilical vein endothelial cells including 200 *μ*L blank serum obtained from control rats, while cells in ox-LDL + medicated serum (MS), ox-LDL + MS + miR-30b-5p-mimic, and ox-LDL + MS + miR-30b-5p-inhibitor were cultured in complete medium for human umbilical vein endothelial cells including 200 *μ*L medicated serum obtained from rats in Zhilong Huoxue Tongyu capsule group described in Section 2.3 above. Cells in control group and ox-LDL group were cultured in complete medium for human umbilical vein endothelial cells supplied with 200 *μ*L phosphate buffer saline (PBS, 201201A17, Yuanmu Biological Technology, Shanghai, China). Next, cells in ox-LDL + MS + miR-30b-5p-mimic and ox-LDL + MS + miR-30b-5p-inhibitor groups were transfected 5 nM miR-30b-5p-mimic and miR-30b-5p-inhibitor, respectively. Cells were maintained at 37°C with 5% CO_2_ for further 24 h.

### 2.6. Cell Counting Kit-8 Assay

HCAECs were inoculated in 96-well plates with a density of 1 × 10^5^/well and cultured for 24 h at 37°C in 5% CO_2_. Subsequently, the Cell Count Kit-8 (Dojindo Laboratories, Kumamoto, Japan) was used to detect the proliferation of cells according to the operating manual. The absorbance was recorded at 450 nm by a microplate reader (Thermo Fisher Scientific, Waltham, MA, USA).

### 2.7. Flow Cytometry

The pyroptosis of HCAECs was determined using pyroptosis/Caspase 1 cell pyroptosis detection kit (9145, ImmunoChemistry Technologies, USA). Cells were collected and resuspended with 500 *μ*l Binding Buffer and then stained with a mixture of 5 *μ*l FAM-YVAD-FMK-FLICA and 5 *μ*l propidium iodide (PI) (Sigma-Aldrich) for 15 min at room temperature in the dark. The rate of pyroptosis was examined by flow cytometry (BD FACSVerse, USA).

### 2.8. Quantitative Reverse Transcriptase-Polymerase Chain Reaction (qRT-PCR)

Total RNA was extracted from cell samples using TRIzol reagent (TaKaRa Biotechnology Co., Ltd., Dalian, China) according to the manufacturer's specifications. cDNA was synthesized with a PrimeScript RT Reagent Kit (Takara, RR047A) in line with the manufacturer's instruction. qRT-PCR was carried out by the Bio-Rad Scrip™ cDNA Synthesis Kit (Bio-Rad Laboratories, Inc., Hercules, CA, USA). The primer sequences were designed and synthesized. The qRT-PCR amplification conditions were as follows: 95°C for 5 min, 95°C for 15 s, and 60°C for 30 s of 40 cycles. *β*-actin served as the internal control. For the quantification of miR-30b-5p expression, the RT reaction was performed using Bulge-Loop™ miRNA RT-qPCR Primer (RiboBio Co., Ltd., Guangzhou, China). The RT reaction was processed at 42°C for 60 min and at 70°C for 10 min. The miR-30b-5p expression level was quantified at 95°C for 10 min, followed by 40 cycles at 95°C for 2 sec, 60°C for 20 sec, and 70°C for 10 sec. U6 served as the internal control. The level of genes was analyzed by the comparative threshold cycle method (2^−ΔΔCT^ method), where ΔΔCT = ΔCT_treatment_ − ΔCT_control_ and ΔCT = *Ct*_target_ − *Ct*_reference_. The primer sequences used in the present study are listed as follows: NLRP3: forward primer: 5′-TCTCAGCACCAACCAGAGCCTCAC-3′, reverse primer: 5′-CCACGCACAGCAGTCTGACTCCAA-3′; ASC: forward primer: 5′-TGGACCAACACAGGCAAGCACTCA-3′, reverse primer: 5′-AGGTCAGGTTCCAGGCTGGAGCAA-3′; Caspase 1: forward primer: 5′-CCTGGCAGGAATTCTGGAGCTTCAATC-3′, reverse primer: 5′-GGCAAGACGTGTACGAGTGGTTGTATT-3′; IL-1*β*: forward primer: 5′-ATCCTCTCCAGTCAGGCTTCCTTGTG -3′, reverse primer: 5′-AGCTCTTGTCGAGATGCTGCTGTGA-3′; IL-18: forward primer: 5′-TGCCTGATATCGACCGAACAGCCAAC -3′, reverse primer: 5′-ACAGATAGGGTCACAGCCAGTCCTCT-3′; *β*-actin: forward primer: 5′-CTGGCACCACACCTTCTACA-3′, reverse primer: 5′-GGGTCATCTTCTCACGGTTG-3′; miR-30b-5p: forward primer: 5′-ACGGGCAAAAATACTCCAGCTCTCAAT-3′, reverse primer: 5′- CTCTGGAAAACTGGTGTCGACTGGTGTC-3′; and U6: forward primer: 5′-GCTTCGGCAGCACATATACTAAAAT -3′, reverse primer: 5′-CGCTTCACGAATTTGCGTGTCAT -3′.

### 2.9. Detection of LDH

The content of LDH was detected using LDH assay kit (A020-1, NJJCBIO, Nanjing, China) according to the operating manual. The absorbance was recorded at 440 nm by a microplate reader (Thermo Fisher Scientific).

### 2.10. Luciferase Assay

Wild-type and mutant NLRP3 were constructed into pGL3-Basic luciferase vector (Promega, Madison, WI, USA) to produce luciferase reporter plasmids. HCAECs were transfected with the plasmids and then were cotransfected with miR-NC or miR-30b-5p mimic with Lipofectamine 2000 (Life Technologies, New York, USA). The luciferase activity was determined by Promega Kit (Promega) after cells were transfected for 48 h.

### 2.11. Statistical Analysis

Data were shown as the means ± SE. Shapiro–Wilk test was employed to test continuous quantitative data distribution normality. Student's *t*-test was employed to analyze the data with only two groups, whereas the one-way analysis of variance was used to analyze the differences among multiple groups by the SPSS 22.0 statistical software (IBM, Armonk, New York, USA) followed by post hoc Bonferroni test. The differences were regarded as statistically significant when *p* < 0.05.

## 3. Results

### 3.1. The Effect of Medicated Serum of Zhilong Huoxue Tongyu Capsule on HCAECs Viability

**Figure 1:**
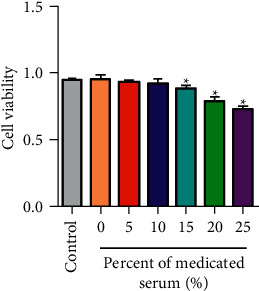
The effect of medicated serum of Zhilong Huoxue Tongyu capsule on HCAECs viability. HCAECs were hatched with different concentrations of medicated serum of Zhilong Huoxue Tongyu capsule for 24 h (0, 5, 10, 15, 20, and 25%). The cell viability was detected by CCK-8 Kit. Data of four independent samples were shown as mean ± SE. ^*∗*^*P* < 0.05. The medicated serum of Zhilong Huoxue Tongyu capsule relieves the pyroptosis of HCAECs induced by ox-LDL in vitro.

HCAECs were hatched with different concentrations of medicated serum of Zhilong Huoxue Tongyu capsule for 24 h (0, 5, 10, 15, 20, and 25%). The cell viability of HCAECs was dose-dependently reduced with medicated serum of Zhilong Huoxue Tongyu capsule. The maximum nontoxic concentration of medicated serum of Zhilong Huoxue Tongyu capsule was 10% (Figure 1). Thus, 10% medicated serum of Zhilong Huoxue Tongyu capsule was used in subsequent assays.

**Figure 2:**
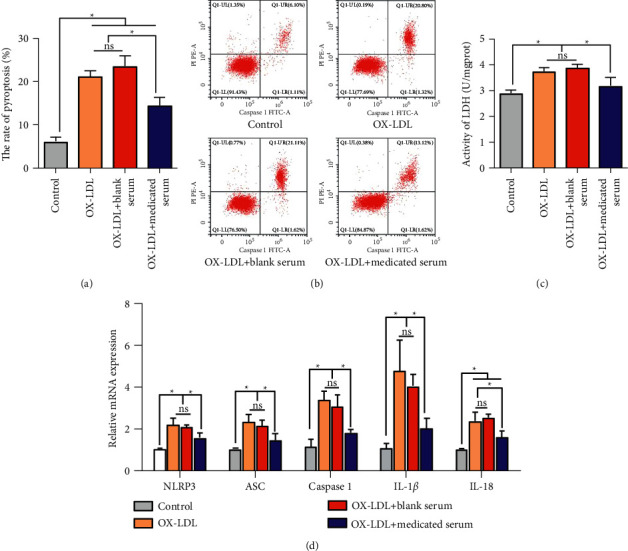
The medicated serum of Zhilong Huoxue Tongyu capsule relieves the pyroptosis of HCAECs induced by ox-LDL in vitro. HCAECs were treated with 50 nM ox-LDL for 24 h to induce atherosclerosis cell models and then incubated with 200 *μ*L Zhilong Huoxue Tongyu capsule medicated serum. (a, b) The rate of pyroptosis was determined by flow cytometry assays. (c) The LDH content was detected using commercial kit. (d) The relative mRNA levels of NLRP3, ASC, Caspase 1, IL-1*β*, and IL-18 were detected by qRT-PCR assay. The data were expressed after being normalized to *β*-actin. Data of three independent samples were shown as mean ± SE. ^*∗*^*P* < 0.05.

Then, in order to evaluate the effect of medicated serum of Zhilong Huoxue Tongyu capsule on the pyroptosis, AS cell models were induced by ox-LDL. Flow cytometry assays exhibited that the rate of pyroptosis was prominently enhanced after HCAECs were induced by ox-LDL, which was markedly rescued with medicated serum of Zhilong Huoxue Tongyu capsule, not blank serum (Figures 2(a) and 2(b)). Similarly, medicated serum of Zhilong Huoxue Tongyu capsule, not blank serum, notably reduced the ox-LDL-induced elevation of LDH content (Figure 2(c)). Besides, qRT-PCR results exhibited that all the mRNA expression levels of pyroptosis-related genes including NLRP3, ASC, Caspase 1, IL-1*β*, and IL-18 were significantly increased after HCAECs were induced by ox-LDL, which was markedly reversed with medicated serum of Zhilong Huoxue Tongyu capsule, not blank serum (Figure 2(d)). Therefore, these results indicated that the medicated serum of Zhilong Huoxue Tongyu capsule relieved the pyroptosis of HCAECs induced by ox-LDL in vitro.

### 3.2. NLRP3 Binds to miR-30b-5p

**Figure 3:**
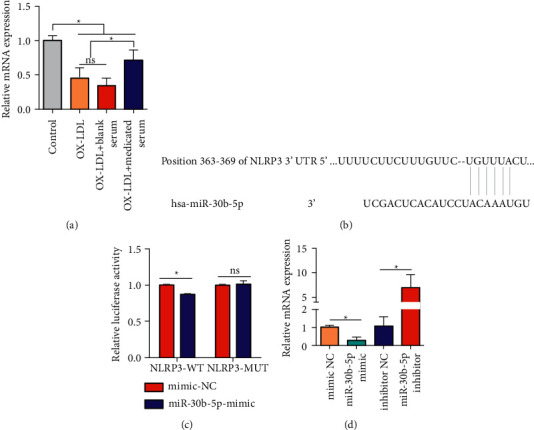
NLRP3 binds to miR-30b-5p. (a) HCAECs were treated with 50 nM ox-LDL for 24 h to induce atherosclerosis cell models and then incubated with 200 *μ*L Zhilong Huoxue Tongyu capsule medicated serum. The level of miR-30b-5p was detected by qRT-PCR assay. The data were expressed after being normalized to U6. (b) The binding sites of miR-30b-5p and NLRP3 were forecasted via the bioinformatics website. NLRP3 wild-type plasmids and mutant plasmids were constructed. (c) NLRP3 wild-type and mutant plasmids were constructed into pGL3-Basic luciferase vector to produce luciferase reporter plasmids. HCAECs were transfected with the plasmids and then were cotransfected with miR-NC or miR-30b-5p mimic with Lipofectamine 2000; after cells were transfected for 48 h, the direct interaction of NLRP3 and miR-30b-5p was verified by luciferase reporter experiment. (d) The relative mRNA level of NLRP3 was detected by qRT-PCR assay after HCAECs were transfected with miR-30b-5p mimic or miR-30b-5p inhibitor. The data were expressed after being normalized to *β*-actin. Data of three independent samples were shown as mean ± SE. ^*∗*^*P* < 0.05.

In addition, the level of miR-30b-5p was significantly reduced after HCAECs were induced by ox-LDL, which was notably antagonized with medicated serum of Zhilong Huoxue Tongyu capsule, not blank serum (Figure 3(a)). To further explore the underlying molecular mechanism of miR-30b-5p, an NLRP3 wild-type plasmid (NLRP3-WT) or an NLRP3 mutant plasmid (NLRP3-MUT) was constructed for the luciferase reporter assay (Figure 3(b)). The results showed that the luciferase activity of NLRP3-WT reporter plasmids was dramatically inhibited compared to that in the negative control (NC) in HCAECs. However, no statistical difference was observed in the luciferase activity of NLRP3 compared to that in the NC in HCAECs (Figure 3(c)). Moreover, miR-30b-5p mimic prominently inhibited the level of miR-30b-5p, while miR-30b-5p inhibitor notably elevated the level of miR-30b-5p (Figure 3(d)). Based on these results, we concluded that NLRP3 could bind to miR-30b-5p.

### 3.3. The Medicated Serum of Zhilong Huoxue Tongyu Capsule Alleviates the Pyroptosis of HCAECs Induced by ox-LDL through miR-30b-5p/NLRP3

**Figure 4:**
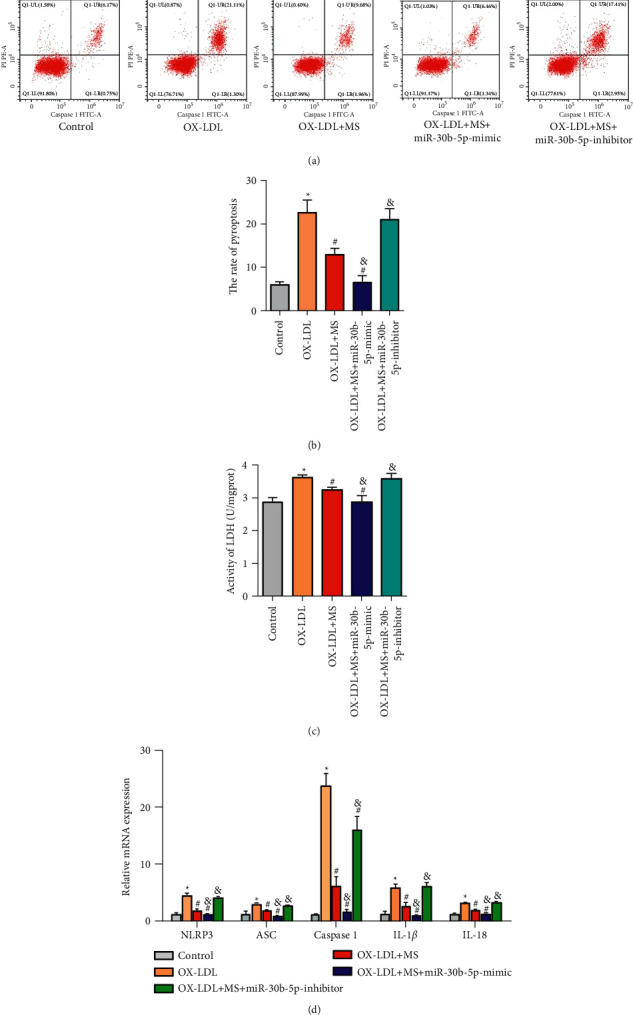
The medicated serum of Zhilong Huoxue Tongyu capsule alleviates the pyroptosis of HCAECs induced by ox-LDL through miR-30b-5p/NLRP3. HCAECs were treated with 50 nM ox-LDL for 24 h to induce atherosclerosis cell models and then incubated with 200 *μ*L Zhilong Huoxue Tongyu capsule medicated serum. Next, cells were transfected with 5 nM miR-30b-5p-mimic and miR-30b-5p-inhibitor, respectively. (a, b) The rate of pyroptosis was determined by flow cytometry assays. (c) The LDH content was detected using commercial kit. (d) The relative mRNA levels of NLRP3, ASC, Caspase 1, IL-1*β*, and IL-18 were detected by qRT-PCR assay. The data were expressed after being normalized to *β*-actin. Data of three independent samples were shown as mean ± SE. ^*∗*^*P* < 0.05 compared to control group. ^#^*P* < 0.05 compared to ox-LDL group. ^&^*PP* < 0.05 compared to ox-LDL + MS. MS: medicated serum.

The pyroptosis of HCAECs was assessed after ox-LDL-induced HCAECs were transfected with miR-30b-5p mimic or miR-30b-5p inhibitor. The results revealed that all the rates of pyroptosis (Figures 4(a) and 4(b)), LDH content (Figure 4(c)), and the mRNA expression levels of pyroptosis-related genes including NLRP3, ASC, Caspase 1, IL-1*β*, and IL-18 (Figure 4(d)) were further observably decreased after ox-LDL-induced HCAECs treated with medicated serum were transfected with miR-30b-5p mimic, while these were significantly rescued with transfection of miR-30b-5p inhibitor. Taken together, these results indicated that the medicated serum of Zhilong Huoxue Tongyu capsule alleviates the pyroptosis of HCAECs induced by ox-LDL through miR-30b-5p/NLRP3.

## 4. Discussion

ox-LDL plays a key role in the pathological process of AS which can act on the vascular endothelium and recruit inflammatory cells, thereby promoting the production of foam cells and the formation of atherosclerotic plaques, leading to the occurrence of endothelial cell pyroptosis [23, 24]. Current lipid-lowering drugs have achieved the effect of effectively reducing LDL, but how to remove ox-LDL is still a difficult problem. Under normal conditions, ox-LDL produced in the body can be quickly cleared by Kupffer cells in the liver [25]. However, continuous lipid metabolism disorder and inflammation in AS lead to increased ox-LDL in the circulation [25]. Therefore, this study mainly explores new therapeutic directions besides lipid lowering and focuses on Zhilong Huoxue Tongyu capsule effectively removing cell pyrosis induced by ox-LDL. Our previous studies have demonstrated a protective role of Zhilong Huoxue Tongyu capsule in the ischemic stroke and related diseases, as well as AS [26, 27].

Modern pharmacology confirmed that the main pharmacological components of *Astragalus* in Zhilong Huoxue Tongyu capsule are *Astragalus* total flavonoids, *Astragalus* polysaccharides, and *Astragalus saponins*. *Astragalus* total flavonoids can scavenge oxygen free radicals in vivo. *Astragalus* polysaccharides (APS) may play a direct anti-inflammatory role, inhibit nuclear factor *κ*B expression, and scavenge free radicals, thereby reducing nerve cell apoptosis and accelerating the recovery of nerve function defects. *Hirudin*, the main component of leech, is the strongest thrombin inhibitor discovered so far. It combines with the active center of thrombin to form a stable noncovalent bond compound, thus inhibiting the hydrolysis of thrombin to fibrinogen and blocking the coagulation cascade reaction. At the same time, the combination of thrombin and *Hirudin* could not lyse the extracellular N-terminal of the receptor activated by protease, so the thrombin receptor could not be activated, thus inhibiting the receptor-mediated neuronal apoptosis and stimulating the activation, proliferation, and secretion of cytokines of microglia [28]. In addition, the collateral circulation can be opened by activating blood circulation and removing blood stasis, so that the hematoma can be in contact with the surrounding blood vessels to promote its absorption and improve the surrounding hypoperfusion injury [29]. In the present study, we established the role of Zhilong Huoxue Tongyu capsule in the pyroptosis. The results revealed that Zhilong Huoxue Tongyu capsule alleviated the pyroptosis of vascular endothelial cells induced by ox-LDL through miR-30b-5p/NLRP3.

AS is a complicated inflammatory disease, in which endothelial cells, macrophages, and vascular smooth muscle cells (VSMCs) are mainly three types of cells involved in its progression [30–32]. Among them, endothelial cells act on a core role in maintaining the integrity of vessel wall, and the injury of endothelial cell is generally regarded as the initiation of AS [33]. Thus, HCAECs were used in the present study. Inflammation and cell death are basic features in the start and development of AS, and pyroptosis has been demonstrated to be a type of inflammatory cell death and involved in a variety of cardiovascular diseases [34, 35]. Moreover, it has been reported that ox-LDL can induce cell pyroptosis in HCAECs [36], macrophage [37], and VSMCs [38]. Consistently, our results showed that ox-LDL induced the pyroptosis of HCAECs, as evidenced by the elevation of pyroptosis rate, LDH content, and the mRNA expressions of NLRP3, ASC, Caspase 1, IL-1*β*, and IL-18. Pyroptosis is a form of Caspase 1-dependent cell death, and the Caspase 1 activation is essential [39]. Also, the activation of Caspase 1 needs the participation of inflammasome including NLRP3, in which NLRP3 recruits ASC and accelerates the activation of Caspase 1 [40]. Upon Caspase 1 activation, it carries out its function to process the precursor of the inflammatory cytokines IL-1*β* and IL-18 to generate their mature forms IL-1*β* and IL-18 [41]. However, the medicated serum of Zhilong Huoxue Tongyu capsule reduced the ox-LDL-induced enhancement of pyroptosis rate, LDH content, and the mRNA expressions of NLRP3, ASC, Caspase 1, IL-1*β*, and IL-18. Therefore, these results indicated that Zhilong Huoxue Tongyu capsule alleviates the pyroptosis of HCAECs induced by ox-LDL.

miRNAs are tightly involved in the progression of ox-LDL-induced AS [42, 43]. Among them, miR-30b-5p is identified to be a possible diagnostic and therapeutic biomarker for AS due to its dysregulation both in vivo [44] and in vitro [45]. We discovered that the level of miR-30b-5p was notably downregulated after HCAECs were treated with ox-LDL, which was prominently rescued by medicated serum of Zhilong Huoxue Tongyu capsule treatment. Thus, the results suggested that Zhilong Huoxue Tongyu capsule alleviates the pyroptosis of HCAECs induced by ox-LDL through upregulating miR-30b-5p expression. The activation of inflammatory response is an important process in the apoptosis and injury of endothelial cells caused by ox-LDL, and ox-LDL-induced activation of inflammatory response can directly cause endothelial sclerosis and thus participate in the formation of atherosclerotic plaques [46]. Meanwhile miR-30b-5p has been implicated in activation of NLRP3 inflammasome in other studies [47]. Importantly, many research studies suggest that NLRP3 is involved in pyrophosis of AS [12, 48]. Besides, ox-LDL induces the activation of NLRP3 inflammasome in the aorta [49], which is important for the formation of unstable plaques in AS by promoting Caspase 1 activation of cell pyroptosis involved in ox-LDL-induced apoptosis [48]. Furthermore, miR-30b-5p is negatively correlated with NLRP3 [21], which was confirmed by our results including the dual-luciferase reporter gene system results and qRT-PCR results. In order to further explore the detailed mechanisms, UVECs were transfected with miR-30b-5p mimic or miR-30b-5p inhibitor. The results revealed that the rate of pyroptosis, LDH content, and the mRNA expression levels of pyroptosis-related genes including NLRP3, ASC, Caspase 1, IL-1*β*, and IL-18 were further observably decreased after ox-LDL-induced HCAECs treated with medicated serum were transfected with miR-30b-5p mimic, while these were significantly rescued with transfection of miR-30b-5p inhibitor. Therefore, these results indicated that Zhilong Huoxue Tongyu capsule alleviates the pyroptosis of HCAECs induced by ox-LDL through miR-30b-5p/NLRP3 and also suggested that the increased expression of miR-30b-5p and the inhibition of NLRP3 inflammasome activation may be the molecular mechanism of Zhilong Huoxue Tongyu capsule to reduce the vascular endothelial cells induced by ox-LDL.

However, combined with the findings of various studies on Zhilong Huoxue Tongyu capsule, there are still some problems that need further discussion. For example, in terms of drug metabolism and pharmacokinetics, there is still a lack of studies on Zhilong Huoxue Tongyu capsule in vivo, which makes it difficult to find its active substance basis for the treatment of cardiovascular diseases. In addition, the basic research of Zhilong Huoxue Tongyu capsule was in-depth research on the mechanism of AS, lacking the understanding of the overall mechanism of action. Therefore, molecular biological techniques such as genomics, proteomics, and metabolomics, combined with the analysis of active ingredients, can be used to further study the mechanism of its action in preventing and treating common cardiovascular diseases, providing a solid basis for its clinical application.

In conclusion, this present in vitro study reports observable effectiveness of Zhilong Huoxue Tongyu capsule on the pyroptosis of vascular endothelial cells induced by ox-LDL. Mechanistically, Zhilong Huoxue Tongyu capsule alleviated the pyroptosis of vascular endothelial cells induced by ox-LDL through miR-30b-5p/NLRP3. Zhilong Huoxue Tongyu capsule can promote the expression of miR-30b-5p and inhibit the activation of NLRP3 inflammasome in vascular endothelial cells induced by ox-LDL. Moreover, miR-30b-5p can directly target NLRP3 in vascular endothelial cells induced by ox-LDL and reduce the activity of NLRP3 inflammasome. The results of this study will provide new insights and methods for the therapy of AS and even other inflammation-related cardiovascular and cerebrovascular diseases.

## Data Availability

The data used or analyzed during the current study are available from the corresponding author.
